# Health and Well-Being Trajectories in Diverse Social Contexts

**DOI:** 10.1093/geroni/igaf122.2077

**Published:** 2025-12-31

**Authors:** Laura Buchinger, Denis Gerstorf, Toni Antonucci

## Abstract

This symposium aims to bring together a collection of papers that consider both social presence and absence, as well as different types of social ties, to provide a nuanced perspective on the role of interpersonal connections for late-life health and well-being. Rauer and colleagues challenge singlehood stereotypes by examining how desire for a romantic partner, gender, and age shape satisfaction with being single. Lewis and colleagues examined the role of co-exercise in enhancing the mood benefits of physical activity. They found that exercising with a close social partner regardless of relationship type amplified the positive effects of physical activity on mood. Birditt and colleagues examined caregiving experiences across relationship types, age groups, and dementia caregiving status, finding that younger spousal caregivers for individuals with dementia experience the highest strain. Buchinger and colleagues analyzed the longitudinal links between physical health constraints and perceived stress, finding that maintaining strong social ties can mitigate stress associated with frailty and functional decline. The studies employ rigorous methodologies, leveraging large-scale nationally representative data, advanced statistical modeling, daily life ambulatory assessments, and the integration of self-reported and objective measures. They examine multiple dimensions of health and well-being, including subjective well-being, physician-assessed physical health, and health behavior. Together, these studies underscore the critical role of social contexts in shaping psychological and physical health across adulthood and late life. Discussant Toni Antonucci will provide an integrative perspective on the broader implications for social and aging research.

